# No Evidence of a Causal Relationship between Plasma Homocysteine and Type 2 Diabetes: A Mendelian Randomization Study

**DOI:** 10.3389/fcvm.2015.00011

**Published:** 2015-03-05

**Authors:** Jitender Kumar, Erik Ingelsson, Lars Lind, Tove Fall

**Affiliations:** ^1^Molecular Epidemiology and Science for Life Laboratory, Department of Medical Sciences, Uppsala University, Uppsala, Sweden; ^2^Cardiovascular Epidemiology, Department of Medical Sciences, Uppsala University, Uppsala, Sweden

**Keywords:** homocysteine, type 2 diabetes, Mendelian randomization, MTHFR, association

## Abstract

**Background:** Several observational studies have shown an association between increased circulating homocysteine and risk of type 2 diabetes (T2D). We aimed to assess whether this relation is causal using genetic data from large populations of individuals of European descent.

**Methods:** We investigated the association between homocysteine concentrations and blood glucose, plasma insulin, T2D in the Prospective Investigation of the Vasculature in Uppsala Seniors (PIVUS) cohort (*n* = 1,016). A score of five previously published single nucleotide polymorphisms (SNPs) from genes involved in homocysteine metabolism were utilized as genetic instrument for homocysteine concentrations. The effect estimate of this genetic score with T2D was determined using results from the DIAbetes Genetics Replication And Meta-analysis (DIAGRAM) consortium (including 34,840 cases and 114,981 controls). Further, the effects of the genetic score with fasting glucose and insulin were determined using results from the Meta-Analyses of Glucose and Insulin-related traits Consortium (MAGIC) (up to 38,238 non-diabetic participants).

**Results:** The genetic score provided a strong instrument for homocysteine concentrations (*P* = 2.7 × 10^−143^, *F* = 650). In the PIVUS cohort, we found an association of homocysteine with fasting insulin [β = 0.056 (95% CI 0.021, 0.090), *P* = 0.001], but not with incident diabetes. We did not find any evidence of a causal effect of homocysteine on fasting glucose, fasting insulin, or T2D (*P* > 0.05 for all analyses) when using data from DIAGRAM or MAGIC studies.

**Conclusion:** No evidence of a causal relationship of levels of plasma homocysteine with fasting glucose, fasting insulin, or T2D was observed.

## Introduction

Type 2 diabetes (T2D) is one of the most prevalent metabolic diseases and has already reached epidemic proportion in many countries (1). A multitude of factors (both genetic and environmental) contribute to the pathogenesis of T2D (2–4). If not treated adequately, eventual outcomes due to diabetic complications may be potentially devastating as almost 75% of T2D patients die due to cardiovascular complications (5).

Homocysteine (Hcy) is a sulfur-containing amino acid and is generated from the breakdown of the dietary amino acid methionine (6). Different enzymes, encoded by genes in the Hcy metabolism pathway, play an important role in regulating the levels of Hcy. Several candidate and genome-wide association studies have shown a number of single nucleotide polymorphisms (SNPs) associated with modulated levels of Hcy, many of these within or close to genes implicated in the Hcy metabolic pathway (6–11). Further, dietary factors such as vitamin B12 and folate also play important roles in maintaining optimum level of Hcy (6).

Elevated level of Hcy (hyperhomocysteinemia) has been shown to be a risk factor for cardiovascular diseases and T2D development (12, 13). Many studies have also shown association between increased levels of Hcy with T2D-associated features such as impaired beta-cell function and insulin resistance (3, 14, 15). These adverse outcomes in response to hyperhomocysteinemia have been suggested to be related to various mechanisms such as oxidative stress and inflammation, contributing components of T2D pathogenesis (6, 16).

In Mendelian randomization (MR) study designs, one or several genetic variants, usually SNPs, associated with exposure to a modifiable risk factor are used as instrumental variables (IV) to provide unbiased estimates of the causal relationship of the exposure to the risk factor (here Hcy) with an outcome of interest (here T2D and related traits) (17). Previous MR studies have provided conflicting results about causal effect of Hcy on T2D (18–20).

We applied an MR framework utilizing results from the genome-wide association study (GWAS) meta-analysis including up to 34,840 T2D cases (21) and up to 46,186 individuals of European descent (22) with T2D intermediate trait measures to assess the potential causal relationship between Hcy and diabetes and related traits. We used data from a population-based study to compare estimates derived from causal analyses with estimates derived from conventional analyses.

## Materials and Methods

### Materials

#### Prospective Investigation of the Vasculature in Uppsala Seniors

We used data from the Prospective Investigation of the Vasculature in Uppsala Seniors (PIVUS) study for assessing the association of Hcy with diabetes and diabetes-related traits. The PIVUS study has been described in detail elsewhere (23). Briefly, all subjects aged 70 years living in Uppsala, Sweden were eligible. The subjects were chosen from the community register and invited in a randomized order from the start of the study in April 2001 to the last included subject in June 2004. Of the 2,025 subjects invited, 1,016 subjects (507 men and 509 women) agreed to participate and were included in the study. All the participants gave their written informed consent and the Ethics committee of Uppsala University, Uppsala approved the study. Participants underwent health assessment including questionnaires, clinical examinations, and a detailed biochemical panel. Participants underwent subsequent examinations at ages 75 and 80 years. During the visit at age 80 years, all medical records were screened for diagnosis of diabetes. Biochemical profiling of blood samples collected at baseline (age 70 years) was performed at the University Hospital, Uppsala, Sweden. Plasma insulin was measured with a chemiluminescence assay (Roche, Basel, Switzerland). Fasting blood glucose was measured in whole blood utilizing HemoCue instrument (HemoCue, Ängelholm, Sweden) following manufacturer’s recommendation and recalibrated to plasma concentrations by multiplying with a factor of 1.11. Diabetes was defined as a doctor-diagnosed history of diabetes or a fasting blood glucose ≥7.0 mmol/l at the examination. Level of Hcy was measured using AxisVR Homocysteine Enzyme Immunoassay (Axis-Shield Diagnostics).

#### Genetic data

To create genetic instruments, we used results from the largest GWAS of Hcy levels based on meta-analysis of 44,147 individuals of European descent (11). In order to achieve a specific instrument and minimize the risk of pleiotropic effects, we excluded all SNPs mapped to genes outside the Hcy metabolism pathway, e.g., hepatocyte nuclear factor 1 homeobox A (*HNF1A*). Therefore, the final selection included five SNPs from three loci: 5,10-methylenetetrahydrofolate reductase (*MTHFR*), 5-methyltetrahydrofolate-homocysteine methyltransferase (*MTR*), and cystathionine-beta-synthase (*CBS*), where *MTHFR* and *CBS* contained two independent signals each. We assessed the results for the five selected SNPs from van Meurs et al., where effects (β) of each SNP were reported in the per-allele effect on lnHcy (SD-units). The effect estimates of each of the Hcy SNPs on T2D and diabetes-related traits were collected from GWAS meta-analysis results – the largest studies available with GWAS data. The effect on T2D risk was estimated by utilizing a study by DIAbetes Genetics Replication And Meta-analysis (DIAGRAM) consortium including 34,840 cases and 114,981 controls (21). We transformed reported odds ratios for T2D to lnOR scale for further analysis. The effect estimate of each Hcy SNP on measures of glucose homeostasis was based on associations with fasting glucose in up to 46,186 non-diabetic participants and fasting insulin in up to 38,238 non-diabetic participants from the Meta-Analyses of Glucose and Insulin-related traits Consortium (MAGIC) (22). Effects were reported on non-transformed scale for fasting glucose and on log-transformed scale for fasting insulin. The effect of each SNP is reported in Table 1.

**Table 1:** **Association of Hcy SNPs with diabetes and related traits, based on large genome-wide association studies**.

				ln Homocysteine			T2D			Fasting glucose			ln Fasting insulin		
SNP Gene	rsID	EA	EAF	β	SE	*P*	β (lnOR)	SE	*P*	β	SE	*P*	β	SE	*P*
*MTHFR*	rs12134663	C	0.20	0.10	0.01	2.5 × 10^−21^	0.01	0.02	0.78	0.002	0.005	0.69	−0.002	0.005	0.63
*MTHFR*	rs1801133	A	0.34	0.16	0.01	4.3 × 10^−104^	0.02	0.01	0.10	−0.003	0.004	0.46	−0.005	0.004	0.25
*MTR*	rs2275565	G	0.79	0.05	0.01	2.0 × 10^−10^	0.00	0.02	0.93	−0.001	0.005	0.91	−0.003	0.005	0.50
*CBS*	rs234709	C	0.55	0.07	0.01	3.9 × 10^−24^	0.01	0.02	0.57	0.003	0.004	0.38	0.003	0.004	0.51
*CBS*	rs2851391	T	0.47	0.06	0.01	1.7 × 10^−12^	−0.02	0.02	0.32	−0.001	0.004	0.87	−0.002	0.004	0.66
SCORE				0.09	0.004	2.7 × 10^−143^	0.008	0.008	0.34	0.0002	0.002	0.90	−0.002	0.002	0.38

*T2D, type 2 diabetes; SNP, single nucleotide polymorphism; MTHFR, methylenetetrahydrofolate reductase; MTR, 5-methyltetrahydrofolate-homocysteine methyltransferase; CBS, cystathionine-beta-synthase; EA, effect allele; EAF, effect allele frequency; β, beta; SE, standard error; *P*, significance; ln(OR), natural log of odds ratio*.

### Statistical analysis

#### The association of Hcy plasma concentrations with T2D, fasting insulin, and fasting glucose

For all analyses in PIVUS, Hcy was transformed to the natural logarithmic scale and thereafter SD transformed. We assessed the association of Hcy with ln-transformed insulin and glucose in non-diabetic subjects from PIVUS using linear regression modeling with glycemic traits as the dependent variable adjusting for age and sex. We used a logistic regression model to assess the potential association with prevalent diabetes at baseline adjusting for age and sex. We assessed the association of Hcy with incident T2D using Cox proportional hazard models for time-to-T2D adjusting for age and sex. Subjects who died during the study period were censored. The assumption of proportional hazards was assessed by testing the associations of Schoenfeld residuals and time. We also performed secondary analysis in a subset of PIVUS samples where we tested whether adjusting for intake of vitamin B12 and folate were confounders of Hcy with prevalent (*n* = 853) and incident diabetes outcome (*n* = 750).

#### Association of genetic score with levels of Hcy

We identified the Hcy-increasing allele of each SNP in the data together with its reported effect from van Meurs et al. (11) and used the R-package “gtx” to estimate the combined effect of the genetic score (β_score_Hcy_) on levels of Hcy based on the summary level data. Briefly, β_score_Hcy_ was calculated as $\sum \beta_{i}s_{i}^{-2}∕\sum s_{i}^{-2},$ where β_i_ was the effect of the Hcy-increasing risk alleles on Hcy and *s*_i_ its corresponding standard error (24). The output of the equation gives an estimate of the per-allele increase in lnHcy of the genetic score.

#### Association of genetic score with T2D, fasting insulin, and fasting glucose

All analyses were performed using the Hcy-increasing allele as the effect allele. The effect of the non-weighted genetic score (β_score_outcome_) on the outcomes of interest: T2D, fasting glucose, and fasting insulin, respectively, was calculated as $\sum \beta_{i}s_{i}^{-2}∕\sum s_{i}^{-2},$ where β_i_ is the effect of the Hcy-increasing risk alleles on T2D or T2D-related trait and *s*_i_ its corresponding standard error using the R-package “gtx.”

#### Instrumental variable analyses

We used IV estimators to quantify the strength of causal association between Hcy and T2D, fasting glucose, and insulin. The estimator (β_IV estimator_) was found as a ratio between two regression coefficients determined from the genetic score analysis (Eq. 1): estimated genetic score on given outcome (β_score_outcome_) and estimated genetic score effect on Hcy (β_score_Hcy_).

(1) $$\beta_{\mathrm{IV estimator}}=\frac{\beta_{\mathrm{genetic instrument\_outcome}}}{\beta_{\mathrm{genetic instrument\_Hcy}}}$$

The standard errors (SE) for the IV estimators were calculated using the delta method (Eq. 2), which we have previously evaluated for this purpose (25).

(2) $$SE_{\mathrm{IV}}=\mathrm{abs}\left(\beta_{\mathrm{IV}}\right)\times \sqrt{{\left(\;\frac{SE_{\mathrm{genetic instrument\_Hcy}}}{\beta_{\mathrm{genetic instrument\_Hcy}}}\;\right)}^{2}\;\;+{\left(\;\frac{SE_{\mathrm{genetic instrument\_outcome}}}{\beta_{\mathrm{genetic instrument\_outcome}}}\;\right)}^{2}}$$

## Results

The median age of study participants was 70.2 years and almost equal percentage of females and males were present.

### The association between levels of plasma Hcy and T2D, fasting insulin, and fasting glucose

The baseline characteristics of PIVUS participants are shown in Table 2. A total of 11% individuals were found to have hyperhomocysteinemia (levels of Hcy >15.0 μmol/L). One-hundred nineteen individuals had T2D at baseline. Seventy-two individuals developed T2D during 10 years follow-up. In our PIVUS cohort, we found no association between levels of Hcy at age 70 years with prevalent diabetes [odds ratio per SD-unit in ln-transformed Hcy, OR 0.98 (95% CI 0.80, 1.19), *P* = 0.80], incident T2D [hazard ratio, HR 0.79 (95% CI 0.62, 1.004), *P*-value 0.054], or with fasting glucose [β = −0.001 (95% CI −0.038, 0.036), *P*-value 0.96]. Further adjustment in the prevalent and incident diabetes models for intake of vitamin B12 and folate did not change the effect estimates of Hcy. A significant association was observed between levels of Hcy and ln fasting insulin [β = 0.056 (95% CI 0.021, 0.090), *P-*value 0.001], indicating that 1-SD-unit increase in the level of lnHcy was associated with 5.6% (*e*^0.056^) increase in fasting insulin (Table 3).

**Table 2:** **Baseline characteristics of the PIVUS cohort (*n* = 1,016)**.

Variable	Median (IQR) or number (%)
**Clinical variables**
Age (years)	70.2 (70.0, 70.3)
Sex (female)	509 (50.2)
Smokers (current)	107 (10.6)
BMI (kg/m^2^)	26.6 (24.0, 29.7)
WHR (cm)	0.90 (0.86, 0.95)
SBP (mmHg)	148 (134, 164)
DBP (mmHg)	78 (72, 86)
Homocysteine (μmol/L)	9.9 (8.0–12.3)
Glucose (mmol/L)	5.0 (4.6, 5.4)
Insulin (mIU/L)	7.4 (5.2–10.9)
Vit B12 (μg/day)^a^	6.0 (4.6–7.9)
Vit B6 (mg/day)^a^	1.8 (1.5–2.1)
Folate (μg/day)^a^	226 (186–271)
LDL (mmol/L)	3.3 (2.8, 3.9)
HDL (mmol/L)	1.4 (1.2, 1.8)
TG (mmol/L)	1.15 (0.87, 1.51)
GFR (mL/min/1.73m^2^)^b^	78.9 (65.4, 94.9)
**Exercise habits**
No	114 (11.5)
Low	583 (59.0)
Medium	222 (22.5)
High	69 (7.0)
**Education**
Low	569 (56.7)
Medium	182 (18.1)
High	253 (25.2)

*Number (%) is shown for discrete variables while median (IQR) is shown for continuous variables*.

*^a^Dietary intake is shown*.

*^b^Log-transformed value is reported*.

*IQR, interquartile range; BMI, body mass index; WHR, waist–hip ratio; SBP, systolic blood pressure; DBP, diastolic blood pressure; Vit B12, Vitamin B12; Vit B6, Vitamin B6; LDL, low-density lipoprotein cholesterol; HDL, high-density lipoprotein cholesterol; TG, triglycerides; GFR, glomerular filtration rate*.

**Table 3:** **The association of homocysteine with diabetes and related traits in the PIVUS cohort and in causal analysis using large genome-wide association studies**.

	Prevalent T2D			Incident T2D			Fasting glucose			Fasting insulin (ln-transformed)		
	OR	SE	*P*	HR	SE	*P*	β	SE	*P*	β	SE	*P*
PIVUS	0.98	0.10	0.80	0.79	0.10	0.054	−0.001	0.02	0.96	0.05	0.02	0.001
IV estimate	1.01	0.09	0.34				0.0002	0.02	0.90	−0.002	0.02	0.38

*The effect is given per SD-increase in log-transformed homocysteine*.

*OR, odds ratio; SE, standard error; HR, hazard ratio; *P*, significance; β, beta; IV, instrument variable*.

### The association between genetic score and levels of plasma Hcy

We found a strong association between the genetic score and levels of plasma Hcy using the published genetic associations (11). We estimated that each allele of the score increased lnHcy with 0.092 SD (95% CI: 0.084, 0.100; *P*-value 2.7 × 10^−143^). The calculated *F*-statistics for the score was 650.

### The association between genetic score and T2D, fasting insulin, and fasting glucose

We did not find evidence for an association of the genetic score with T2D, fasting insulin, or fasting glucose. The estimated odds ratio for each additional allele of the genetic score was 1.01 (95% CI 0.99, 1.024; *P*-value 0.34) for T2D. The estimated per-allele effect of the genetic score on ln fasting insulin was −0.002 (95% CI −0.006, 0.002; *P*-value 0.38) and 0.0002 (95% CI −0.003, 0.004; *P*-value 0.90) for fasting glucose (Table 3).

### Instrumental variable analyses

We did not find evidence for a causal association of levels of Hcy with T2D and related traits. The estimated causal odds ratio for each SD-increase in lnHcy was 1.09 (95% CI 0.92, 1.30; *P*-value 0.34) for T2D. The estimated causal effect for each SD-increase of Hcy on ln fasting insulin was −0.019 (95% CI −0.060, 0.023; *P*-value 0.335) and 0.002 (95% CI −0.037, 0.042; *P*-value 1) for fasting glucose (Table 3).

## Discussion

In the present study, we did not observe any evidence of a causal relationship between genetically predicted homocysteine concentrations and T2D development. To the best of our knowledge, we have included the largest number of individuals with data on the effect of Hcy-related SNPs on fasting glucose, fasting insulin, and T2D.

### Comparison with previous studies

#### Type 2 diabetes

Several observational studies have found hyperhomocysteinemia to be a risk factor for T2D and related complications (3, 15, 26). However, the results are contradictory and some studies have shown a null effect (27–29). Our observational data from PIVUS did not show evidence of increased risk for T2D with increasing levels of Hcy. On the contrary, our effect estimate was negative, although non-significant (*P* = 0.054). In a large, long-term randomized clinical trial by Song et al. involving 4,252 women at high risk for cardiovascular diseases, lowering Hcy levels through vitamin B6, B12, and folic acid did not reduce the risk of developing T2D (30). Another possibility to evaluate the possible causal connection of Hcy with T2D is the MR approach, where the association of Hcy-related genetic variants with T2D is evaluated, aiming to assess life-long exposure to augmented levels of Hcy. A recent MR study by Zhu et al. assessed whether the *MTHFR* C677T polymorphism is a risk factor for T2D in Chinese Han populations (19). The authors meta-analyzed 29 different case–control studies involving 4,656 T2D cases and 2,127 controls from Chinese population. They have found *MTHFR* C677T polymorphism to be a risk factor for T2D and thereby shown evidence in favor of a causal relation between Hcy and T2D. The variant allele (T) in *MTHFR* gene leads to the formation of thermolabile enzyme with reduced activity thereby increasing levels of Hcy (31). The distribution of *MTHFR* C677T polymorphism differs worldwide with Chinese having higher T allele frequency as compared to Europeans, Africans, or other Asian populations, such as Japanese and Indians (8, 32). Since the frequency of this polymorphism varies even within China, the samples were separated into two major groups (northern and southern) based on their region, but similar association results between the polymorphism and T2D were obtained. In a study by Huang et al., the authors collected data on the *MTHFR* C677T polymorphism, Hcy, and T2D in 4,011 T2D cases and 4,303 controls from published studies (20). The pooled data from 17 studies from different countries showed a significant association of *MTHFR* C677T polymorphism with levels of Hcy, as well as with T2D. However, the data were pooled for different ethnicities together and the authors reported significant heterogeneity among different study groups involved in meta-analysis. In another study by Zhong et al. involving 4,855 diabetic patients and 5,242 controls from different ethnic backgrounds (Asians, Europeans, and Africans), no association between the Hcy-increasing SNP *MTHFR* C677T and T2D was observed (18). Even in the repeated meta-analysis including individuals only from same ethnic group, similar results were obtained for Europeans and other populations thereby corroborating our results. The conflicting results obtained in our study as compared to the Chinese study have several possible explanations. Ethnic background of the study subjects is different and there can be variation in gene–environment interaction due to several environmental factors.

#### Fasting insulin

We observed a significant association between measured levels of plasma Hcy and fasting insulin in the PIVUS study. Several observational studies have shown association between Hcy and insulin resistance (3, 26, 33). In the Framingham offspring study, Meigs et al. studied 2,011 individuals without CVD or T2D, and observed significant association between hyperhomocysteinemia and hyperinsulinemia (3). The results obtained in the causal part of our study do not support the causal relation between homocysteine and fasting insulin concentrations, which indicates that the observed association between Hcy and insulin in PIVUS and other studies may be explained by either reverse causation or residual confounding.

#### Fasting glucose

We did not observe an association between level of Hcy and blood glucose, in the observational analyses in PIVUS, or in the IV analyses using large-scale data from the MAGIC consortium. Several other observational studies have been performed to understand the relation between Hcy and glucose (34–38). Our study corroborates the results obtained in earlier studies.

#### Strengths and limitations

The strengths of this study are the inclusion of a large number of individuals and the use of multiple SNPs from Hcy metabolism pathway in the causal analysis, which increases the statistical power of our study. To the best of our knowledge, this is the first MR study that has comprehensively evaluated the association of multiple SNPs from Hcy metabolism pathway with levels of fasting glucose, fasting insulin and T2D in large number of individuals. Although genetic variants in the gene hepatocyte nuclear factor 1-alpha (HNF1A) are associated with Hcy, we chose not to include it in the genetic IV, because of its function in the regulating expression of several liver and pancreatic-islet specific genes and its association with MODY type 3. Due to the regulating action of this gene on several other genes, pleiotropic effects may not be ruled out thereby making this gene unfavorable for inclusion into MR studies. Nevertheless, our study also have potential limitations, which are mainly connected to the validity of the assumptions underlying the causal interpretation within MR. There are three main assumptions for a MR study: (1) independence between the instrument and confounders, i.e., genotypes are randomized; (2) a reliable association between the genetic variant and intermediate phenotype; and (3) conditional independence between the genetic variant and the outcome, given the intermediate phenotype and the confounders, i.e., no pleiotropy. Neither the first nor the third assumption can be tested statistically in the observed data using single genotypes as IV, and conclusions about these have to be based on previous biological knowledge. In the present study, we have used only robustly associated SNPs from large GWAS that reside in genes from the Hcy metabolic pathway, and we therefore regard the instrument as specific and non-pleiotropic. The random distribution of genotypes in the population is the very basis of MR and could be violated if separate ethnic groups with different allele frequencies were analyzed together without accounting for the population substructure. In the present study, all association analysis was done using published GWAS of individuals of European descent. In these studies, extensive work has been made to identify and correct for population stratification. Concerning the reliability of the second assumption (association between genetic score and Hcy), the strength of the association was high.

## Conclusion

In conclusion, although a strong association of Hcy with fasting insulin was noted in non-diabetics in observational analyses, we did not find any evidence of a causal link between Hcy with insulin, glucose, or T2D.

## Conflict of Interest Statement

The authors declare that the research was conducted in the absence of any commercial or financial relationships that could be construed as a potential conflict of interest.
